# A Risk-Based IoT Decision-Making Framework Based on Literature Review with Human Activity Recognition Case Studies

**DOI:** 10.3390/s21134504

**Published:** 2021-06-30

**Authors:** Tazar Hussain, Chris Nugent, Adrian Moore, Jun Liu, Alfie Beard

**Affiliations:** 1 School of Computing, Ulster University, Jordanstown, Co. Antrim BT37 0QB, UK; cd.nugent@ulster.ac.uk (C.N.); aa.moore@ulster.ac.uk (A.M.); j.liu@ulster.ac.uk (J.L.); 2 BT Labs, Adastral Park, Martlesham Heath, Ipswich IP5 3RE, UK; alfie.beard@bt.com

**Keywords:** IoT framework, machine learning, uncertainty, domain knowledge, human activity recognition

## Abstract

The Internet of Things (IoT) is a key and growing technology for many critical real-life applications, where it can be used to improve decision making. The existence of several sources of uncertainty in the IoT infrastructure, however, can lead decision makers into taking inappropriate actions. The present work focuses on proposing a risk-based IoT decision-making framework in order to effectively manage uncertainties in addition to integrating domain knowledge in the decision-making process. A structured literature review of the risks and sources of uncertainty in IoT decision-making systems is the basis for the development of the framework and Human Activity Recognition (HAR) case studies. More specifically, as one of the main targeted challenges, the potential sources of uncertainties in an IoT framework, at different levels of abstraction, are firstly reviewed and then summarized. The modules included in the framework are detailed, with the main focus given to a novel risk-based analytics module, where an ensemble-based data analytic approach, called Calibrated Random Forest (CRF), is proposed to extract useful information while quantifying and managing the uncertainty associated with predictions, by using confidence scores. Its output is subsequently integrated with domain knowledge-based action rules to perform decision making in a cost-sensitive and rational manner. The proposed CRF method is firstly evaluated and demonstrated on a HAR scenario in a Smart Home environment in case study I and is further evaluated and illustrated with a remote health monitoring scenario for a diabetes use case in case study II. The experimental results indicate that using the framework’s raw sensor data can be converted into meaningful actions despite several sources of uncertainty. The comparison of the proposed framework to existing approaches highlights the key metrics that make decision making more rational and transparent.

## 1. Introduction

Internet of Things (IoT) refers to a network of all physical objects (things) interacting and sharing information via machine-to-machine (M2M) communications [1,2]. IoT is the enabling technology for many real-life applications, e.g., transportation, healthcare, energy management and agriculture [2,3]. In an IoT setup, a large number of raw data are collected from different types of sensors and are then transmitted for further processing through long- and short-range communication technologies [2,4]. The processed data are analyzed to identify the high-level context and infer useful knowledge, which can then be used to make critical decisions via actuators. However, the existence of several sources of uncertainty is a key challenge that has significant impacts on decision making in IoT systems [5]. Uncertainty is a general term that is used to describe concepts like incompleteness, imprecision and ambiguity [6]. The existence of uncertainty may lead decision makers to take inappropriate actions that might have significant impacts, especially in cases where IoT systems are associated with high-risk decisions [7]. The sources of uncertainty in IoT-based decision-making include uncertainties associated with data acquisition, data processing, data analysis and incomplete coverage of a specific domain [8].

In IoT systems, these uncertainties may be caused by the availability of limited battery and processing power, high security and information exchange requirements, etc. [9]. The complexity of an IoT system increases along with different characteristics of the system, such as heterogeneity, scalability, dynamism and timeliness; these factors make it even harder to manage uncertainty. 

In an IoT-based Decision Support System (DSS), Machine Learning (ML) algorithms are used to identify high-level situations and discover useful knowledge based on historical data [10]. However, ML algorithms face several key challenges to handle uncertainties in their predictions [11]. For example, supervised ML algorithms entirely rely on the training data, which might be incomplete and imprecise due to sensor noise or intermittent network connection problems [12]. In addition, the absence of an adequate number of training data and imbalanced class distributions has a great impact on the performance of the ML algorithms. Normally, the level of confidence associated with a prediction via ML is not quantified; that is, standard ML algorithms provide only crisp outputs. However, in high-risk IoT setup situations, it is vital to quantify the confidence associated with each prediction made via ML and take them into consideration, which may, in turn, affect the final critical decision making to a large extent [13].

Motivated by the aforementioned challenges, in the present work, we aim to propose a risk-based IoT decision-making framework for the Human Activity Recognition (HAR) domain. The objective of the presented work is to reduce the negative impact of uncertainties, minimize risk and improve decision-making in HAR. The framework is “risk-based” in the sense that it provides a mechanism to prioritize critical IoT functions and treat an uncertainty as a risk only when it affects the objectives of the HAR use case. The proposed framework is essentially an integration of data-driven and knowledge-based approaches, which provides an interesting framework capable of bridging lower-level activity recognition and higher-level knowledge for explanation and recommendation, as well as handling uncertainties. The proposed framework is expected to enhance decision making in HAR-based applications despite the existence of various sources of uncertainty. An IoT-based HAR system is the requirement of many real-life applications such as pervasive computing, remote health monitoring, smart home environments and Ambient Assistive Living (AAL) [14]. In these examples, there is great potential to reduce strain on healthcare and hospital resources. It could also be used to monitor non-critical patients at home or those living in rural areas and can enable elderly people to live more independently [3]. 

In a real-life HAR decision-making application, an ML algorithm must not only be accurate in predicting user’s activities, but it should also provide indications when it is incorrect. For example, in a HAR-based automated health care system, human intervention is required if a critical health-related activity is predicted below a specified threshold. Therefore, the ML algorithm should provide a reliable confidence score in its prediction, which means that the predicted probability should reflect the true probability. Therefore, a new ensemble-based random forest algorithm, called a Calibrated Random Forest (CRF), is proposed to handle uncertainty and acquire more reliable predictions, in situations where a class probability estimation for each prediction is used to quantify uncertainty in the CRF model’s predictions. A probability calibration technique is implemented to produce well-calibrated class probability estimations so that these estimations can be directly used as the confidence scores associated with the prediction via ML.

In the present work, an openly available HAR dataset [15] is used to evaluate the framework and various types of uncertainties have been identified. A remote health monitoring scenario has been developed to monitor the activities of a user with a lifestyle disease (diabetes in this case). The related activities that influence diabetes from the dataset have been identified, and the idea is that if a specific activity deviates from its normal frequency, it can be declared as a symptom of diabetes. Similarly, for remote monitoring applications, this example scenario depends on accurate predictions from the underlying IoT infrastructure. Therefore, activities have been identified with confidence scores and context in order to monitor the user and take appropriate actions. 

Generally, the proposed framework offers several key benefits to manage uncertainties and make the decision process more rational in a HAR-based health-related use case. The specific contributions of this paper are several-fold; firstly, we propose a CRF model and combine the resulting confidence scores of the CRF with the HAR domain knowledge and contextual information. Secondly, both the domain knowledge and prediction confidence scores are encoded in IF-THEN rules to support decision making in the diabetes scenario. Thirdly, the framework proposes a natural way to integrate domain knowledge; e.g., a threshold defined by the domain expert has been set, and predictions made above that threshold are trusted. The framework also provides a mechanism to interpret the model’s behavior and infers situations that can be recognized easily by medical experts. The results indicate that the framework is able to identify reliable situations that can be automated in the HAR-based DSS. On the other hand, situations with confidence less than a threshold are forwarded for human investigation. Finally, based on the inferred situations, appropriate actions are recommended. A comparison with the existing approach reveals that the proposed framework provides a mechanism to make decision making more rational despite the existence of uncertainties in the IoT setup. 

The remainder of the paper is organized as follows. Section 2 provides a brief review of related work. Section 3 presents the details of the proposed framework. The details of the dataset used in this study are presented in Section 4. Section 4 also presents and discusses the results of the proposed CRF method. Section 5 provides details of a diabetes scenario to evaluate the CRF model. Furthermore, Section 6 analyzes and compares the overall result of the framework. Finally, Section 7 concludes the work and identifies some future directions.

## 2. Related Works

IoT is an enabling technology to support situation awareness and decision making in many real-life applications. However, researchers have identified uncertainty in both the physical world and in IoT technology as one of the key challenges that limit its application to support decision-making [3,10]. The quality of data has also been identified as a significant element for IoT-based decision making in supply chain management [16]. The literature has argued that for IoT systems to make intelligent decisions the interaction and communication alone are not enough. The “things” must have the capability to learn, think and understand; this concept is known as cognitive IoT [17]. In this study, decision making with uncertain and incomplete information has been described as the key challenge in building cognitive IoT. Researchers in [7,18] have also identified heterogeneity, weak semantics, limited resource availability and sensor network malfunction as the key sources of imprecise, inaccurate and incomplete data in IoT applications. Therefore, a data semantization [19] technique has been proposed as the key solution to address the challenges posed by IoT systems. In this approach, domain knowledge information is used to add semantics to IoT data for making more informed and intelligent decisions. Similarly, in many IoT systems, sensor data is uncertain due to several reasons, and decision support systems greatly rely on such data for automated decision making [18,20]. In previous studies, different approaches have been devised to reduce the impact of uncertainties and reduce risks [21,22,23]. For example, a framework architecture based on domain ontology and reasoning systems has been presented to resolve uncertainties [12]. The work presented in [9] analyzed the IoT characteristics, structure and behavior as sources of uncertainty in the context of smart buildings. The study identifies some key strategies to overcome uncertainty including differentiating between significant and insignificant data, rational planning of business strategy and continuously updating the developing scenario. A novel IoT medical DSS has been proposed to detect and monitor diabetes patients [24], where sensors are used to collect blood pressure, heart rate, respiratory rate and glucose to determine the symptoms. The study handles ambiguity and incompleteness with an intuitionistic fuzzy set.

The knowledge-driven approaches make use of rich domain knowledge in IoT systems to build models using knowledge engineering and representation methods. These knowledge engineering techniques formalize the domain knowledge using logical reasoning and ontology-based methods. Another way to exploit the rich domain knowledge is to develop a rule-based system. For example, a rule-based finite state machine method has been applied to recognize activities from binary sensors [25]. The results indicate that the approach outperforms other data-driven approaches. However, one of the drawbacks of these knowledge-driven approaches is the lack of a mechanism to handle uncertainty explicitly. Therefore, several studies have proposed to handle uncertainty in the HAR domain; for example, the authors of [26,27] have proposed a solution applying Dempster-Shafer (DS) theory to manage uncertain data generated by sensors. The benefits of using DS theory provide an opportunity to infer context for an activity even if some sensor data are noisy or missing. Researchers have exploited the availability of rich domain knowledge to address shortcomings of insufficient data, as described in [25]. The benefit of using knowledge-driven approaches is that these techniques do not require training and can be used immediately after deployment. However, these knowledge-based techniques do not adapt well to the dynamic situations in the HAR environment [14].

Several data-driven ML algorithms, such as Naïve Bayes Classifiers (NBC), Decision Trees (DT), Neural Networks (NN) and Random Forest algorithms (RF), are used to learn patterns from sensor data and predict an activity [28,29]. These ML algorithms offer some important benefits for IoT applications including dynamic adaptation and uncertainty handling as studied in [11]. ML approaches have also been used for making important decisions in critical scenarios [2,30]. However, ML-based techniques are limited to model randomness and variability as specific types of uncertainty. For critical applications, the lack of a mechanism to quantify uncertainty in ML output has been identified [13,31] as an essential obstacle to making reliable decisions in an IoT setup. In addition, researchers [21,32] have identified the limited availability of training data and incomplete and imprecise sensor data as the main drawbacks of a data-driven approach. Therefore, to improve the quality of the training data, researchers have applied ontological concepts to raw sensor data to enhance ML performance. 

Furthermore, probabilistic ML algorithms offer some key benefits for managing uncertainty in prediction models and improving decision making, as discussed in [33]. A probability calibration technique has been proposed [34] for calibrating classifier probability scores to the probability of disease level in order to develop uncertainty estimates in clinical decision support systems. A probability calibration workflow for making cost-sensitive classification decisions has been proposed in [35]. The approach minimizes the cost of a prediction instead of improving the performance. Similarly, the authors of [36] have proposed techniques for binary classification that outperform traditional probability calibration techniques for binary classifiers. Similarly, in the proposed work in this paper, these techniques also focus on improving the calibration of the model. However, these techniques lack a mechanism to integrate domain knowledge with probability calibration metrics, such as thresholding and cost of actions, in cases of making high-risk decisions. Therefore, the proposed work not only quantifies uncertainty in a ML output but also integrates domain knowledge with these ML outputs to manage risk in critical decision-making scenarios. 

Another approach to handle uncertainty is the deployment of a hybrid approach [37,38], which has been implemented and integrates ML with a fuzzy ontology. A similar study [11] has extended the domain knowledge approach as input to the ML algorithm to improve situational awareness. Although these hybrid techniques integrate domain knowledge with ML, they do not provide a mechanism for managing the risk associated with actions in a specific scenario. On the contrary, they propose approaches for integrating domain knowledge with actions to better handle risk and criticality. As it can be noted from the current literature, various studies have implemented different techniques to address the risks associated with decision making in IoT. Additionally, these techniques have been implemented in an isolated manner. Nevertheless, our proposed framework is distinct from existing approaches, as it is more structured and explicitly addresses the risks associated with taking actions by integrating domain knowledge with machine learning. The other unique features of the framework include quantifying uncertainty in ML outputs and bringing humans in the loop to deal with more critical situations.

## 3. A HAR Risk-Based IoT Decision Making Framework

This section presents an overview of the proposed framework, its comparison with a standardized IoT architecture is also analyzed, and then components and subcomponents of the IoT-DM framework are explained. 

### 3.1. Framework Overview

**Figure 1:**
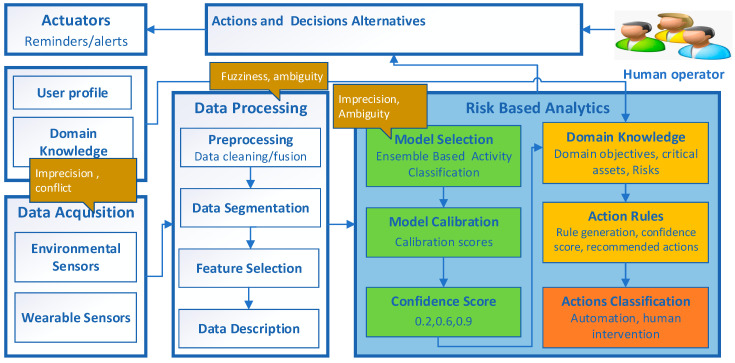
The proposed IoT-DM framework.

The proposed risk-based IoT decision-making (denoted as IoT-DM) framework is described in Figure 1 and is designed for use in a HAR domain to convert the raw sensor data into executable actions that can be used in a real-life scenario. IoT-DM is a high-level model that outlines the basic building blocks of IoT systems and highlights the inter-relationships between these building blocks. The modular structure of the framework is designed to achieve the objective of converting raw sensor data into meaningful actions. The data acquisition and processing modules enable the collection of data from heterogeneous sensors and the preparation of these data for processing. The risk-based analytics module extracts useful information using ML, and subsequently, the inferred knowledge is used for decision making. This module is also responsible for quantifying the impact of uncertainties in the data analytics process. Action rules are utilized to combine the ML output with domain knowledge based on the scenario requirements as shown in Figure 1, where the data analytics via ML and domain knowledge subcomponents are represented with green and yellow colors, respectively, while the orange color represents the output action. The golden color bubbles indicate the types of uncertainties involved in data acquisition, processing, and analytics. Subsequently, based on the level of risk, actions are taken to trigger actuators to achieve the required objectives. The human operator is included to monitor the current situations, infer possible future implications and make decisions that require nuance and judgment. The final decisions are either implemented in an automatic manner or through human intervention.

### 3.2. Mapping IoT-DM Framework to Standard IoT Architecture

**Figure 2:**
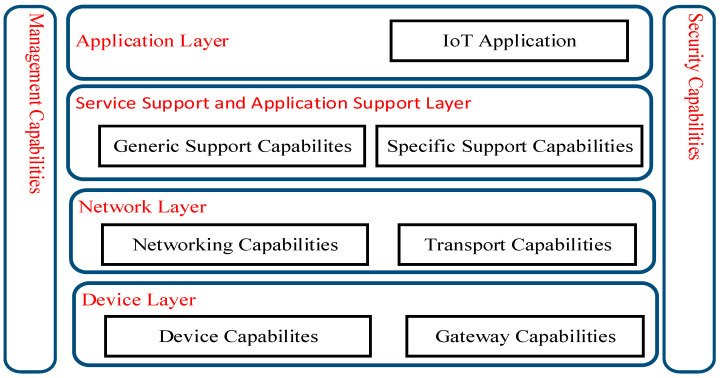
ITU–T IoT Reference Model [39].

Although the proposed IoT-DM framework is deployed for a HAR based e-health use case in this study, it can also be built into common application support platforms providing specific services such as model calibration, domain knowledge and action rules generation. ITU–T (International Telecommunication Union–Telecommunication) is responsible for issuing recommendations for standardizing telecommunications systems worldwide [39]. The IoT reference model from ITU–T is a standardized four-layer architecture that facilitates the IoT implementation for specific systems and use cases. Therefore, we have mapped the components of the IoT-DM to the ITU–T standardized reference architecture to facilitate interoperability and simplify and ease development [40]. The ITU–T reference model is composed of four high-level layers and two layers associated with these four layers as shown in Figure 2.

Table 1 shows the mapping of components from the proposed IoT-DM to the ITU–T layers. The application layer contains an IoT application. The components of the IoT-DM framework such as the Action and Decision Alternative module and its interface to the human operator are mapped to this layer. The service support and application support layer consists of two sub-layers. Generic support capabilities are the common services such as data processing and data storage that can be used by different IoT applications. Furthermore, the specific support capabilities sublayer, which deals with the specific requirement of different IoT applications, is the most relevant layer as most of the IoT-DM components are mapped to generic and specific capabilities as elaborated in Table 1. The services under specific capabilities can be used for critical applications where reliability in decision making is required. 

Network layer capabilities facilitate both common network connectivity functions and transport of specific IoT application information. Although there are no explicit components from IoT-DM that can be mapped to the network layer, the communication flow (indicated by arrows) between IoT-DM components is mapped to the network layer. The device layer provides support for devices to communicate directly with the communication network via an ad hoc networking facility. This layer supports indirect communication of devices (wearable, environmental sensors and actuator) through gateway capabilities using ZigBee, Wi-Fi and 3G technology protocols. 

Similarly, the management capabilities layer covers fault management, configuration management and performance management across all four layers. The security capabilities layer provides authorization, authentication and data confidentiality services to all four layers. There are no components of IoT-DM that can be mapped to the management and security capabilities layers of the ITU–T framework. Therefore, this limitation of the IoT-DM framework must be taken into consideration where these capabilities are critical for an application.

### 3.3. Components of IoT-DM Framework and Sources of Uncertainty

This section presents a brief overview of components of the IoT-DM framework and sources of uncertainty associated with these components. 

#### 3.3.1. Data Acquisition and Processing and Sources of Uncertainty

Data acquisition is the process of collecting a large number of data from different types of sensors, e.g., wearable and environmental sensors [41]. However, the collected sensor data might be incomplete, imprecise and inaccurate due to sensor noise, transmission errors and network connection problems [12]. Data from IoT sensors come in a variety of data types to perceive the status of “things” ranging from numbers to strings. Heterogeneous sensor data increase the chances of errors and make it difficult to combine data with different formats [18]. The collected raw data are normally of weak semantics and need to be combined with other sources of information, e.g., contextual information to support high-level applications [7].

Data processing involves data cleaning and combining data from different sources in a process known as data fusion [42]. Data cleaning requires removing irrelevant and duplicated data while missing data are inserted. Feature selection is the process of identifying a relevant set of features and removing redundant features. Both data fusion and feature selection involve fuzziness and ambiguity [8] as it is challenging to decide whether a particular feature belongs to a certain subset. Similarly, it is also hard to select an optimum set of features with comparatively more discriminative power. 

#### 3.3.2. Data Analytics and Sources of Uncertainty

After data are acquired and processed, data analysis techniques are used to extract useful knowledge, which is then used to make informed decisions [12]. There are multiple aspects of data analytics encompassing diverse techniques and approaches. ML is the technique of learning from experience without being explicitly programmed [2]. Supervised ML techniques (also known as models) use data to learn patterns from; the learned model is then used to predict unobserved data—also known as the testing data [14]. However, the existence of various types of uncertainties makes it challenging to use predictions made by the ML techniques for precise decision making. An ML technique learns from the training data; therefore, any potential noise or imprecision in the training data or in the feature selection process will impact its learning ability and its performance on the testing data [11]. Likewise, in supervised ML techniques, training data are annotated, and it is very likely that an instance or observation is assigned to an incorrect class by an error known as labeling noise or class noise [43]. One of the sources of uncertainty in ML is also the imbalanced data in a classification, referring to a condition when classes are not equally represented in the training data for a specific domain [44]. In such situations, ML techniques normally generalize well for the majority class while it becomes difficult to predict the minority class accurately. 

Due to the above uncertainties, models make imperfect predictions, and as a result, such predictions are quite different from what is expected, as each ML algorithm learns from data and performs an optimization to maximize a likelihood (or posterior distribution) and minimize the error (or loss function). As a result of this optimization, standard ML algorithms predict a crisp output $\bar{y}$ [13]. However, in critical application scenarios, it is crucial to quantify uncertainty, that, is to quantify a model’s confidence in its prediction. For example, it is critical in an AAL application to accurately identify the medication activity as it might involve taking a critical action about the health of the user, e.g., disabling a reminder service.

### 3.4. Risk-Based Analytics

This section illustrates the key components and sub-components of the risk-based analytics module of the IoT-DM framework. 

#### 3.4.1. Ensemble-Based Data Analytics

One limitation of traditional ML techniques such as DT, SVM, LR and NB for classification is their lower performance in comparison to ensemble techniques. On the other hand, deep learning (DL) techniques normally achieve good performance. However, these techniques are more complex and require a significant amount of data for training to achieve good results. Additionally, DL also lacks transparency as the predictions are not explainable to a non-technical user which is required in our case. 

To trade off the benefits and drawbacks of different ML techniques, RF is the more suitable option as it offers some key advantages in our case. RF is an ensemble ML algorithm that uses the bootstrap technique to make a prediction from multiple decision trees; this technique reduces the high variance of individual decision trees and thus avoids overfitting [45]. We have used this technique in the HAR domain as it helps in reducing the variance if there is a change in the training data due to a change in sensor values. One of the challenges of the HAR domain is that similar events are generated for similar activities, and it is difficult for ML techniques to discriminate between such activities. To overcome this challenge, RF generates a subsample from the training data and then fits a single decision tree on each subsample. The predictions from each tree are combined to make the final prediction through a majority vote. Moreover, RF is a suitable option because in this work we require reliable probability estimates to generate a confidence score. As the RF algorithm combines results from a number of decision trees, the probability assigned to each prediction is more reliable than a single decision tree algorithm. 

#### 3.4.2. Probability Calibration to Manage Uncertainty 

In ML, instead of predicting a crisp class as output, several classification algorithms predict the probability of an observation (instance) belonging to each possible class. Such classifiers predict conditional probability distributions as P(X|Y); for a given sample P(X|Y), it assigns an output y ϵ Y [46,47], as shown in Equation (1):

(1) $$\bar{y}=\arg\underset{y}{\max}=P(Y=y|X)$$

The class with the highest probability is the predicted class $\bar{y}$ and is presented as the final (crisp) output. NB, DT, SVM, LR and RF are examples of probabilistic ML algorithms [48], which normally provide probability distributions of predication.

##### Calibration Techniques

Although an ML algorithm may be able to provide a probability distribution of predication, the predicted probabilities may not match the observed probability or the true probability of an event. Therefore, the predicted probabilities cannot be used directly as the confidence scores. Instead, the predicted probability can be modified to better represent the true probability estimates [47]. The process of adjusting the predicted probability is known as calibration. The goal of the calibration technique is to minimize the difference between the predicted and true probabilities.

(2) $$\overset{´}{p}\left(y|x\right)=p(y|x)$$

$\overset{´}{p}$ is the predicted probability of an ML algorithm for an outcome given x, and p is the true probability. As indicated in Equation (2), equal probabilities mean a perfect calibration. 

Certain models, such as LR, inherently generate well-calibrated probabilities as they optimize the conditional probability P(X|Y) (also known as log loss) directly in the training phase and therefore usually do not require calibration [33]. However, many classification algorithms including NB classifier, DT and RF algorithms generate misleading probability distributions and therefore require calibration. The DT algorithm generates distorted probabilities due to high bias and high variance when it tries to produce pure leaves. NB assumes feature independence and forces probabilities to extremes (either 1 or 0) [49]. As RF uses multiple trees and it is difficult for all trees to generate 0 or 1 probabilities, since these trees are trained on different subsets of data, the RF algorithm pushes the probabilities to the center (away from either 0 or 1) as it averages the probabilities from multiple trees with high variance.

In high-risk IoT applications, where risk is involved with decision making, it is important to see the confidence score of the prediction. Ultimately, decision making will take this confidence score of the prediction into account. The mistakes predicted with high probability (overconfidence) and correct prediction with low probabilities (under confidence) can be investigated through domain knowledge.

Platt Scaling (Sigmoid) and Isotonic Regression (IR) are the two techniques commonly used for probability calibration [50,51]. Platt Scaling fits a logistic regression model on the probability estimates generated by an algorithm such as RF to achieve calibration. Platt Scaling generates probabilities in the range of [0,1] and is directly applicable to binary class problems but can be extended to multiclass problems using a one-vs.-rest approach. Although originally proposed to scale the output of an SVM, it also produces good results for boosted models and NB Classifiers [46].

IR is a useful non-parametric calibration technique for many classification models [50]. This technique does not assume anything about the mapping function of a model and therefore performs better than Platt Scaling in several cases. The performance of IR is not constrained by the mapping function of a model as long as the function is monotonically increasing. Both Platt Scaling and IR use a separate validation set or cross-validation to avoid overfitting. This means that the data used for calibration should not be used for training the model [46]. 

###### Evaluation of Calibration 

In binary class, probability calibration can be evaluated using the reliability diagram. This diagram plots the true probability against the predicted probability of a model [51]. However, reliability diagrams are not suitable for multiclass setups. Therefore, log loss and Brier score are proposed as the scoring rules to evaluate the calibration of ML models in a multiclass scenario.

Log loss is commonly used as a scoring rule to evaluate a model’s uncertainty [52]. The distance between the actual and predicted probabilities is measured on a logarithmic scale and is then used to penalize the probability. The higher the distance between the actual and predicted probabilities, the higher the loss, and a log loss of 0 indicates perfect calibration. The log loss for a single sample is calculated as:

(3) −log P(yt|yp)=−(yt log(yp)+(1−yt) log(1−yp))

where yt is the true label and is represented by either 0 or 1 and yp is the estimated probability when yt is equal to a true class or 1. The Brier score is a scoring metric that evaluates the predicted probabilities of a model [53]. It computes the mean squared error between predicted probabilities and the actual probabilities and can be calculated for multiclass problems as:

(4) $$\frac{1}{N}\;\overset{N}{\underset{t=1}{\sum }}\;\overset{R}{\underset{i=1}{\sum }}\;{\left(fti-oti\right)}^{2}$$

Similarly, to log loss, the brier score is always between 0 and 1, where a score of 0 indicates perfect calibration. Well-calibrated probabilities can be interpreted as confidence levels of an ML algorithm. 

#### 3.4.3. Probability Calibration and Rational Decision Making

Predicting calibrated probabilities offers some key benefits to making IoT application decision making more rational and cost-sensitive. Probability calibration provides a method to set a threshold, and only predictions made above that threshold can be trusted to make a rational decision in a given scenario [54,55]. Well-calibrated probabilities also make decision making cost-sensitive by calculating the expected cost of taking an action. For example, if $L\left(k,\overset{´}{k}\right)$ is the cost or the loss function and y=k is the true prediction, and the ML algorithm predicts $\overset{´}{k}$, then the expected cost associated with decision making can be calculated as follows:

(5) $$\sum^{\;}k\;P\left(y=k|x\right)\;L\;\left(k,\;\overset{´}{k}\right)$$

If we obtain a well-calibrated estimation, then a better estimate of the loss ($\overset{´}{k}$) can be attained to minimize the expected loss associated with taking an action. The loss or cost associated with making a decision can also be minimized by estimating the cost of abstention, L(k,abstain). 

Well-calibrated probability estimates provide a way of interpretation in HAR, such that an activity can be interpreted in human language. In some cases, probability calibration can improve the accuracy of a classifier by adjusting the probability threshold used by the classifier for splitting data.

### 3.5. Integration of Data Analytics and Domain Knowledge for IoT Systems

In an IoT setup, ML algorithms mostly rely on learning from data, yet data are not the only source of learning. In addition, some domain knowledge is nearly always available along with the data [56]. Although domain knowledge is used implicitly as part of the ML process (e.g., feature engineering), it is required to get insights into the specific domain explicitly. It has become important in IoT systems to acquire domain knowledge, e.g., to identify objectives and understand the context of the problem and potential solutions. Uncertainties that impact the objectives of a specific domain are known as risks [57], and uncertainties should not be treated equally. There might be many sources of uncertainties in IoT-based systems but it does not necessarily mean a high risk, since a real risk to society and individuals might exist only in few critical cases. In HAR-based AAL, the domain knowledge included is to identify contextual information, e.g., the name, description, duration, time, location and aim of an activity performed by the user [41]. Furthermore, it is also required to identify low-level information, e.g., the sequence and transitional state of an activity, and high-level information, e.g., the objective and criticality of an activity in a given scenario. 

In IoT infrastructure, one of the major sources of uncertainty is the incomplete coverage of domain information [10] as it is not possible to cover every aspect of the domain. For example, HAR is the domain that involves learning and identifying complex human behavior consisting of sequential and overlapping actions [42]. Humans perform activities in different ways depending on their personal preferences; for many activities, it is also not clear what the exact start and end times are [58]. Similarly, interleaved activities performed by users in a dynamic manner ultimately lead to ambiguity, and it becomes challenging to infer high-level context. 

Risk analysis in IoT setups is used to identify and understand the value of data, the criticality of operations and the scalability of any failures. The goal is to prioritize valuable data and critical functions so that they can be managed with high standards and special care [59]. To reduce the impact of uncertainties in ML predictions, domain knowledge can be combined with the probability estimates of predictions made by ML algorithms. The proposed framework also provides a mechanism to integrate the domain knowledge with confidence scores obtained from ML Predictions and encode it into IF-THEN action rules. 

### 3.6. Action Rules and Decision Alternatives 

After identifying useful patterns, rules are generated to produce reliable and accurate actions. Such rules encode IF-THEN conditions that are used to trigger recommended actions. The domain knowledge in a given scenario can be modeled through these rules with the probability estimate of prediction. Rules can also be generated to classify the actions that should be sent directly to the decision support system and automated, while certain actions that involve criticality and uncertainty at the same time can be forwarded for human investigation. 

One of the major advantages of IoT systems is in their autonomy, since they can help to make decisions automatically without human intervention. However, from a risk analysis perspective, an IoT system must provide a higher degree of human intervention and control for critical assets and operations to reduce risk.

After perceiving the environment through sensors and identifying associated risks in an IoT setup, the next step is to trigger actions using actuators [41]. When a situation is inferred, for example in HAR, whether a “Medication” activity has taken place or not, then a reminder service (as an actuator) can be enabled or disabled accordingly.

## 4. Case Study I: HAR in the Smart Home

This section details a case study in a Smart Home environment with the focus on evaluating the performance of the proposed CRF model within the IoT-DM framework, using the open-source benchmark dataset. 

### 4.1. UCAmI Data Set

**Figure 3:**
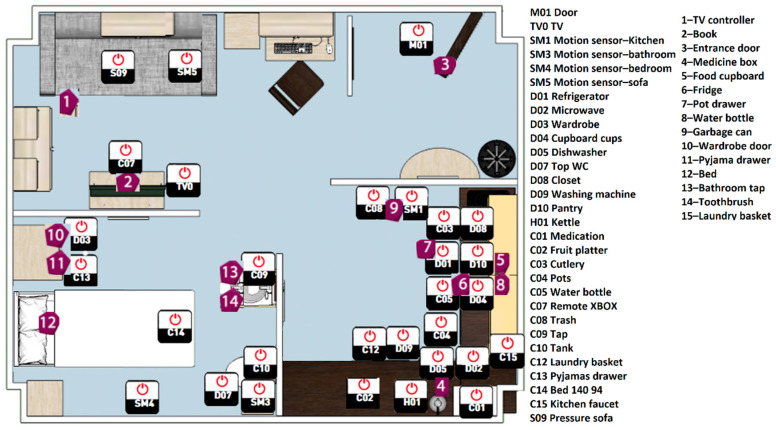
Location of the binary and proximity sensors in the UJAml smart lab.

The dataset used in this case study to assess the proposed framework is from the first UCAmI Cup challenge [15], which was collected by the University of Jaen Ambient Intelligence (UJAmI) smart lab. The researchers were invited to use their tools and techniques to analyze the HAR dataset and present their results. An area of approximately 25 square meters was divided into five regions: entrance, kitchen, living room, workplace and a bedroom with an integrated bathroom, as shown in Figure 3. To capture human–environment interactions, heterogenous data from binary, proximity, accelerometer and floor sensors were used. Figure 3 also shows the location and deployment of the binary and proximity sensors as these two types of sensors have been considered for this study.

A set of 30 binary sensors were deployed, generating binary output together with a timestamp. Three types of binary sensors were used, including magnetic contact, motion and pressure sensors. Magnetic sensors detect the status of an object such as a door, where an “open” message is sent when two pieces of the sensor are separated and a “closed” message is sent when the pieces are put together. Motion sensors detect whether a user has moved in or out of range, and depending on the context, a “Movement” or “No Movement” message is sent. Pressure sensors are attached to textile layers to detect the presence and absence of pressure and generate a “Pressure” or “No Pressure” message accordingly.

A set of 15 BLE sensors were used to record the proximity data and the smart watch worn by the user reads the signals from the BLE beacon and receives the Received Signal Strength (RSSI). A greater value of RSSI received by the smart watch implies a smaller distance between the user and the BLE beacons. As the user goes away from the BLE beacon the value of the RSSI decreases.

A total of 24 activities performed by a 24-year-old male student were captured over a period of 10 days. The user performed 246 instances of activity, and the dataset was partitioned into morning, afternoon and evening sections, as shown in Table 2. The number of instances was obtained for each activity after segmenting them into 5-s windows.

### 4.2. Sources of Uncertainty in UCAmI Dataset

This section presents several sources of uncertainty that were identified by the researchers working with the dataset [25,44], which cause some challenges for the ML algorithms and might impact the accuracy of predictions of an activity, which ultimately might lead the decision makers to make inaccurate decisions.

*High number of classes:* The total number of classes in the dataset is 24, which can be deemed as a high number. As mentioned earlier, in such situations, ML algorithms usually require a large number of data to accurately predict a high number of classes. Similarly, several activities are closely related as they are performed at the same location and have similar sensor value profiles. For example, Act02 (prepare breakfast), Act 03 (prepare lunch) and Act04 (prepare dinner) are performed at the same location with similar sensor reading profiles. The high number of classes was one of the reasons for the low accuracy that was reported by the participant at the UCAmI cup challenge [60]. 

*Imbalanced classes:* In the UCAmI dataset, all the classes are not represented equally, as indicated by the distribution of instances for each class in Table 1. This causes the ML algorithms to favor the majority class and ignore the minority class. In the dataset, some activities such as Act14 (visitor to the smart lab) and Act08 (eat a snack) are underrepresented, with only 14 and 78 instances, respectively. On the other hand, activities like Act03 (prepare lunch) and Act12 (relax on the sofa) are overrepresented classes, with 984 and 859 instances, respectively. 

*Data shifts:* Data shift is a situation when testing and training inputs and outputs have different distributions [61]. In an IoT setup, such situations occur when the sensor data representing a class in the training is quite different from the sensor data in the testing. Data shifts impact the performance of an ML algorithm as they learn from the data and try to predict instances that have not been learned from the training data. This problem becomes more serious when data shift situations exist together with an imbalanced class distribution. This issue was reported by researchers of UCAmI cup challenge as a major factor in the low performance [62]. For example, the input and output distribution of Act09 (watch TV) and Act12 (Relax on the sofa) are quite different in the training and testing data. Furthermore, Act09 and Act12 have imbalanced class distributions in the training and testing sets. 

*Missing data:* In an IoT system, sensor data might not be available due to a malfunction of the sensor or a network connection problem. In UCAmI data, researchers have reported situations where data from the sensors are not available [25]. For example, Act10 (enter smart lab) has been performed by users in the afternoon segment of day 6, yet sensor M01 (door contact sensor) has not been triggered.

*Noise in data:* In the IoT setup, it is common for sensors to send unusual and extreme values, and this may occur due to sensor problems [12]. Upon investigation of the binary sensors, it can be observed that the C14 bed pressure sensor keeps detecting pressure despite the user having left the bed and started another activity. Similarly, in the evening, the TV contact sensor TV0 keeps sending an “open” message even when the user is not watching TV. This may be because the user left the TV remote detached from the second half of the sensor. As described earlier, BLE sensors rely on the RSSI value, where the greater the value of RSSI, the smaller the distance between the user and the BLE beacon. However, due to some factors in the environment, e.g., walls, it might send lower RSSI values and might mistakenly indicate that the user has moved away from the BLE beacon. 

*Labeling errors:* In supervised ML, it is typically required to obtain ground truth and annotate data, as labeling errors (or class noise) occur when an instance is assigned to an incorrect class [56]. In the UCAmI dataset, an example of labeling error is the absence of a label for Act14 (visit the smart lab). On day 3, in the evening segment, the door contact sensor M01 was triggered [25], but Act14 was missed and was not labeled. Similarly, in the day 6, morning section, the binary sensor M01 was triggered with “open” and then “closed” status, and yet no labels are available for the sensor data. However, it is quite possible that the user performed Act13, leave the smart lab, and Act10, enter the smart lab.

*Heterogeneity:* Usually, in an IoT setup, information from one source of sensor is not enough to infer high-level context in intelligent applications such as HAR [7]. Therefore, information from multimodal sensors is combined. As different types of sensors generate data at different frequencies and in different formats, this may give rise to an uncertain situation, as data from different sources are required to be transformed into a single format. For example, in the dataset, it can be observed that binary sensors generate data in binary format, and proximity sensors generate data in numeric format. The process of fusion also impacts the segmentation process and might impact the performance of the ML algorithm if it is not handled with care, as shown in the subsequent section.

**Figure 4:**
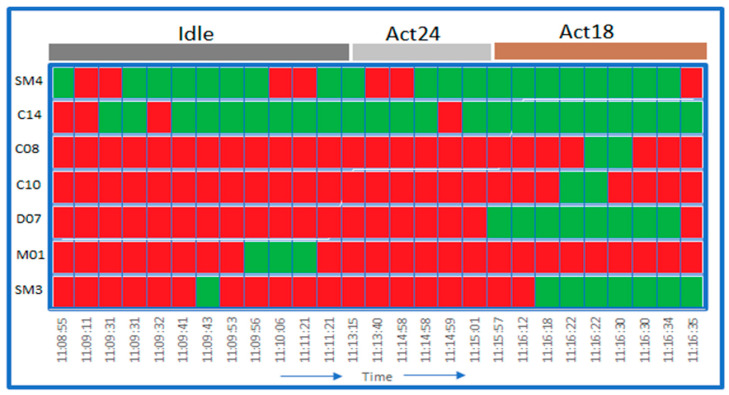
An example of sensor events and activities from the UCAmI dataset morning section of day 1, red and green colors indicate the status of the sensors as OFF and ON respectively.

*Dynamic sensor events:* HAR is usually considered as a classification task; however, some aspects of HAR are not like a simple classification problem. Firstly, HAR generates sensor events in sequence, and as such, these events are not independent [63]. In fact, an event is related to subsequent events in time and space. Secondly, the boundaries between classes are not clear as these activities are similar in function and generate similar sensor events. Additionally, sensor events generated to represent an activity could also represent a transition between two activities. For example, as shown in Figure 4, SM4 (bedroom motion sensor) and C14 (bed pressure sensor) generate very similar events for Act24 (wake up) and Act18 (use the toilet). Although the bathroom motion sensor (SM3) and tank sensor (C10) represent Act18, these sensors generate similar sensor events during the transition from Act24 to Act18. Furthermore, similar sensor events are generated by SM4, C08 (trash sensor) and M01 (door sensor) across idle, Act24 and Act18 activities.

### 4.3. Data Processing

This section presents and explains techniques to prepare UCAmI data for ML algorithms. It includes data pre-processing, data fusion, segmentation and feature selection. 

#### 4.3.1. Data Pre-Processing 

For the purpose of this case study, data from two data sources, namely binary sensors and proximity sensors, were combined. It was also observed that while processing binary sensors, 28 of the 30 sensors were triggered in the dataset. As described earlier, binary sensors and proximity sensors generate data in different formats. As binary sensors generate data in a categorical format, it was decided to represent the binary data as either 1 or 0. For example, the door sensor (M01) was represented as 1 with an “open” status and 0 with a “closed” status. A similar scheme was deployed for all binary sensors and sensor values, with inactive states also represented as 0. On the contrary, BLE sensors generate data in a numeric format in the range of −45 to −106, the data in this range were divided into three groups of low, medium and high, since each group encompasses a range of continuous values and it also helps in reducing the noise caused by environmental factors [64]. As many ML algorithms work only with numeric data instead of categorical data, low, medium and high groups were represented as 1, 2 and 3, respectively. Finally, 64 percent of the data set was selected as the training data and the rest was used as the testing data. 

#### 4.3.2. Data Synchronization

After initial data preparation, the “Timestamp” of each reading from both binary and proximity sensors was aligned and missing data (due to alignment process) were considered as unavailable and represented as 0. As a result of data synchronization, 28 binary sensors and 15 BLE sensors were combined to represent 43 features. Furthermore, each observation was assigned to an activity based on “DateBegin” and “DateEnd”, such that each record represents an activity that was performed during that time. Additionally, a “Time” feature is included to represent morning, afternoon and evening routines with values of 1, 2 and 3, respectively, so that a total of 44 features were generated.

#### 4.3.3. Data Segmentation

The stream of data generated by binary and proximity sensors were segmented into 5-s fixed time windows. In time-based segmentation, the sequence of sensor events is divided into consecutive equal size time intervals [65]. It is crucial to select the optimal segmentation window as it affects the feature selection and the performance of ML algorithms. As the time interval includes sensor events representing the current activity, selecting too-small time intervals might include just a fraction of an activity. On the contrary, large intervals might include sensor events representing several activities. Therefore, experiments were performed with window sizes of 5, 10, 20 and 30 s, and it was found that a 5-s window for binary and proximity data mostly produced good results in terms of accuracy.

#### 4.3.4. Feature Selection

Feature selection is the process of selecting relevant features and removing redundant features. The goal of the feature selection process is to improve the computational efficiency and remove features that might be irrelevant to improve the generalization of the model [41]. A Sequential Forward Selection (SFS) method [66] was applied to select the optimum number of features. SFS is a greedy search algorithm that adds one feature at a time, evaluating the performance of the classifier until the optimal number of features is reached. SFS is a wrapper method of feature selection that uses a model to evaluate and select the best subset of features. The dataset contains 44 features, and most of the values are zeros, such datasets are known as sparse matrices. Generally, a sparse matrix makes inefficient and excessive use of memory. Therefore, SFS is used as it is computationally more efficient because the training is performed on a relatively small subset of features. As shown in Table 3, the number of features was reduced from 44 to 15 using the SFS method, with an accuracy of approximately 86 percent. The RF model was used as the classifier in this case.

### 4.4. Data Analytics for HAR

This section presents the results of the ensemble RF model. This includes the details of the model calibration and its evaluation metrics. Furthermore, the results of the CRF model are explained using feature importance, the classification reported and a confusion matrix.

#### 4.4.1. Calibrated Random Forest (CRF) for Classification 

As proposed in the IoT framework, an ensemble RF algorithm is suggested for class predication, which was implemented using the Python scikit-learn library [48]. Firstly, hyperparameter tuning was carried out to optimize the performance of the RF model using the GridSearchCV() API. A number of experiments were conducted to tune the n_estimators, max_features, max_depth and criterion parameters. However, maximum accuracy (78%) was achieved using the default parameters. Therefore, the default parameters were used with the number of trees set to 100 and the class weights set to “balanced”. In the balanced class weight model, the RF assigns weights to classes inversely proportional to the number of classes in the training data, as explained in the following equation:

(6) $$\omega_{j}=\frac{n}{kn_{j}}$$

where $\omega_{j}$ is the weight of class j, n is the total number of instances, $n_{j}$ is the number of instances in class j, and k is the total number of classes. This measure was taken to address the high imbalanced class distribution in the UCAmI dataset as mentioned earlier. The model has been deployed without any calibration and is referred to as the uncalibrated RF model. The goal of the RF model is to analyze the UCAmI dataset and predict an activity with a probability estimate that represents the true probability of that activity. For this purpose, the scikit-learn API CalibratedClassifierCV was used to implement and compare the state-of-the-art calibration methods. The CalibratedClassifierCV API can be implemented in two ways.

1. A fitted (trained) model is passed, and in this case, the parameter cv is set to “prefit”. In this mode, it is important to keep the data used for training and calibration disjoint to avoid overfitting. Therefore, a separate validation set is required that can be used for calibration only.
2. An unfitted (untrained) model is passed and the parameter cv is set to the number of folds. In this mode one fold is used for training, while subsequent folds are used for calibration.

We have used the API in the second mode as we want to use the maximum number data for training and avoid generating an extra validation set. In this mode, first, an unfitted instance of the RF model was generated with the parameters mentioned above in Section 4.4.1. Subsequently, the RF model was passed as a base estimator to the API, and the cv parameter was set to the default number of folds, i.e., 5. Furthermore, an isotonic regression technique was designed in a one-vs.-rest (OVR) manner for 24 classes. Isotonic regression makes no assumptions about the probabilities generated by the uncalibrated RF and fits a monotonically increasing function for the predicted probabilities. Consequently, the resultant calibrated RF (CRF) is used to predict activities associated with the confidence score, which is useful and will be integrated further with domain knowledge to make decisions. The evaluation and comparison of the CRF has been discussed in the next section. The pseudocode of the CRF is given below.

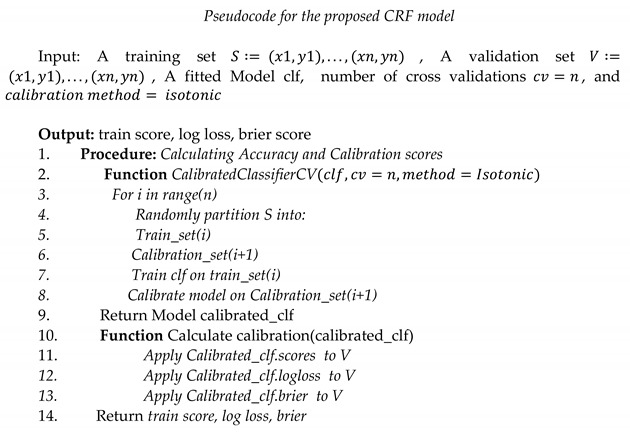


#### 4.4.2. CRF Results Evaluation and Discussion 

**Figure 5:**
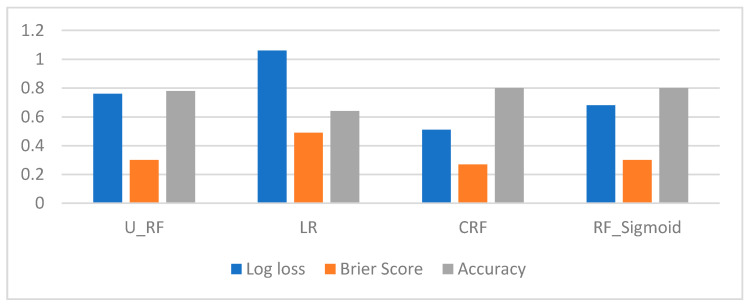
Comparison of the calibration scores and accuracy of the uncalibrated RF, LR and CRF.

The uncertainty evaluation metrics (log loss and Brier score) were used to evaluate the calibration of the CRF model in a multiclass classification setup. The accuracy of the model was also considered for evaluation as it is not always true that better calibration results in greater accuracy. The CRF was compared to other ML models including uncalibrated RF, LR and RF calibrated with sigmoid scaling, and the results are presented in Figure 5. The uncalibrated RF model (represented as U_RF) has achieved an accuracy of 78 percent with a log loss of 0.76 and a Brier score of 0.30. Logistic regression (LR) is considered a benchmark model as it inherently generates well-calibrated probabilities and usually does not require calibration. Therefore, the scikit-learn version of the LR algorithm was deployed to evaluate its performance against the CRF. However, the assessment suggests that LR has performed inadequately with an accuracy of only 64 percent and a high log loss and Brier score of 1.06 and 0.49, respectively. The comparison analysis of the Brier score for the calibrated RF using a sigmoid function indicates no change, yet log loss was reduced to 0.68. Finally, the best calibration and performance were achieved using the CRF with an accuracy of 80 percent, log loss of 0.51 and Brier score of 0.27, which shows a high decrease in log loss and a moderate decrease in Brier score. This is due to the fact that Brier score takes the uncertainty of the data into account and as the level of noise in the dataset is quite high.

Both Brier Score and log loss are methods to evaluate and diagnose the difference between predicted probabilities and true probabilities. The evaluation metrics results indicate that the CRF generates more calibrated probabilities compared to other benchmark ML models. Therefore, the CRF calibrated probabilities lead to better-quality predictions, which can be used as confidence scores for decision making. Furthermore, the CRF confidence scores offer some key benefits for IoT applications as summarized below

- The CRF model does not only indicate when it is accurate, but it also indicates when it is likely to be incorrect in its prediction. For example, the CRF helps to indicate if a critical activity (e.g., medication) has been predicted confidently or not. This property of the CRF helps to improve decision making in real-world applications.
- The CRF enables human operators to use their natural cognitive instruction and interpret the predictions of the RF model. Therefore, the CRF establishes the trust of a user compared to other black-box models, whose predictions are often difficult to interpret. For example, it is quite simple for a human operator to draw different conclusions and subsequently make two different decisions when a medication activity is predicted with a reliability of 0.2 and 0.8.
- The CRF predictions can also be integrated with domain knowledge; for example, a probability distribution of 0.6 and 0.4 represents medication and dinner activities, respectively. This might represent a transition to/from one activity to another.
- The CRF also provides a mechanism to set thresholds for critical activities; for example, a medication activity will be considered reliable only if the predicted probability exceeds 0.8.

## 5. Case Study II: Diabetes Disease Scenario for Demonstration of the IoT Decision Framework

This section presents the details of a diabetes lifestyle disease scenario to demonstrate and evaluate the benefits of the proposed CRF model. Apart from the HAR for identifying diabetes disease, the focus is on evaluating and illustrating how to integrate predictions generated by CRF with the domain knowledge and then provide recommended actions.

### 5.1. Diabetic as a Lifestyle Disease Use Case

HAR could be helpful for caregivers and medical experts in recognizing and monitoring irregular and unhealthy patient activities. Monitoring lifestyle activities can be useful for identifying lifestyle diseases at early stages or can even enable doctors to manage and prevent such diseases [41,67]. One such disease is diabetes, which is associated with symptoms like frequent eating, increased hunger, fatigue, and increased urination. In such chronic diseases, taking medicine on time is also very important for treatment, and an inaccurate identification of medication activity may disable the medication reminder service which could pose a serious threat to the health of the user. Furthermore, in such an application, the goal of the activity recognition algorithm is to identify an activity, such as eating, and if it deviates from a normal frequency (defined by the medical expert), then it can be presented as a symptom of diabetes [67]. The accuracy of such an application entirely depends on the correct identification of an activity. Therefore, it is required to understand both the accurate and inaccurate predictions with their associated confidence scores; otherwise, a misbehaving ML model might lead the medical expert to make an inappropriate decision. For example, if an eating activity is incorrectly identified as sitting, the DSS will identify it as a symptom of diabetes and will mislead the medical experts into making an inaccurate decision. In such a scenario it is not only crucial to rely only on activities that have been identified with a high degree of confidence, but it is also important to recognize incorrect identification of activities due to data noise or classification error. 

In this scenario, we will firstly evaluate the proposed IoT decision framework to identify the symptoms of diabetes and the activities associated with those symptoms. Table 4 shows the symptoms and treatment (medication) of diabetes and their corresponding activities selected from the UCAmI dataset as instances of the breakfast activity (as a type of eating activity).

For the purposes of illustration, a confidence score of 0.8 has been used as a threshold to differentiate more reliable breakfast instances, any instance lower than this set threshold will be analyzed in its context (location, transition and timing) as transition to/from the breakfast activity. Any instance of breakfast activity is identified as noise or prediction error if it is below the threshold and is out of context. 

### 5.2. Rational Decision Making for Diabetes Use Case

This section demonstrates the benefits of the CRF for rational decision making for the diabetes use case. 

#### 5.2.1. High-Risk Decisions Based on the Confidence Threshold

As the medical experts and caregivers use the current activity predicted by an ML algorithm to monitor the status of a user for diabetes, this involves making certain critical decisions (actions) about the health and safety of the user. These critical decisions involve sending reminders for medication, disabling the reminder service, instructions to continue or stop medication or even invoking emergency services if there is a high risk to the user. To make critical decisions in the diabetes use case, an activity predicted by ML with the confidence level provided is far more acceptable or convincing instead of just a crisp prediction under high uncertainties. This means that the medical expert can define thresholds and make decisions based on these thresholds. For example, a threshold (α=0.8) can be set, and any instance predicted to be medication activity with the confidence score above this threshold can be considered as a medication activity; otherwise, further analysis or investigation is required. Table 5 shows examples of predicated and actual instances of the medication activity with the corresponding confidence scores. The “Actual” column represents the ground truth, and the “Predicted” column represents the activity predicted by the CRF model. The rest of the columns represent an activity and its corresponding confidence score. The last column indicates the confidence status of a predicted activity and whether it is above or below the threshold.

The recommended action in such a scenario is to automatically process all predictions of CRF in the decision support system as reliable when the confidence score is above 80% (e.g., the 1st, 3rd, 4th and 7th instances in Table 4). The medication activity or any other activity that is predicted with less than 80% confidence can be further analyzed (e.g., the second, fifth, sixth and nineth instances in Table 4). That is, the following action based on the IF-THEN rule using Equation (2):

Action: ifṕ(ỳ(medication)|x)≥α,thenautomate;elseinvestigate.

The first step in the above analysis, however, is to determine the confidence score of medication and other activities, which is achieved via the CRF.

#### 5.2.2. Cost-Sensitive Decision Making

Decision making in a cost-sensitive way is required when the cost of making a decision is not equal. For example, when y=k and k’ is the model’s prediction, then *L*(*k*, *k*’) is the loss received if y=k. The expected cost of predicting k’ is calculated using Equation (5). Table 6 shows the cost, where a negative cost indicates an instance with the confidence score of a prediction below the threshold.

Similarly, if we want to abstain then L(k,abstain) is the loss if we abstain, while the true answer is k (medication), if (medication (0.7),abstain). In such situations, the loss of abstention is calculated, for example, we can calculate the loss of abstention when the medication activity is predicted with a confidence score of 0.7. This mechanism provides a trade-off between the different actions in situations when the cost of decisions is unequal. 

#### 5.2.3. Interpretability

A probability estimate of the class distribution provides a natural and easy way of interpreting the results. This property of the proposed model enables medical experts and caregivers to understand and predict the cause of a decision made by the model. Table 6 presents an example of instances and their interpretations. The human operator can analyze the situation and take the probability distribution of activities predicted for an instance to form a judgment. For example, it is clear from the interpretability statement of the last instance in Table 7 that the confidence score of the prediction of this medication instance (0.3) is below the threshold. However, from the contextual information (e.g., Act06 0.69 and Act01 0.30 confidence) medical experts can infer that this is in fact the transition to/from a medication activity to an eating lunch activity.

#### 5.2.4. Domain Knowledge Integration 

Similarly, domain knowledge, such as the sequence of activities, locations and times, can be integrated with the probability estimates of a predicted activity in order to improve decision making. As shown in Table 8,

1. Instance numbers 1, 4 and 5 indicate that medication (Act01) has been predicted above the set threshold and has not been confused with another activity. Therefore, these predicted activities are declared as reliable.
2. In Instance 2, the medication activity has been misclassified as Act19 (washing dishes); however, investigating this particular instance indicates that a transition from Act19 to Act01 is actually taking place. Now, taking domain knowledge (transition) into consideration, it can be inferred that the user is coming from Act19 towards Act01 with a confidence of 39 percent.
3. Instance 6 highlights that Act01 has been predicted below the set threshold with a confidence score of 0.51 and Act19 has a probability distribution of 0.38. Therefore, this instance has been inferred as transitional.
4. Instance 7 indicates that Act05 (Breakfast) has a probability of 0.47 and with a 0.52 probability that it is Act02 (Preparing breakfast). Both activities have approximately the same probability, because they take place in the same location (Kitchen) at a similar time in a sequence such that “Preparing breakfast” is followed by the actual “Breakfast” activity. Domain knowledge (location, similar sensor readings, sequence and time) can be combined with the probability distribution of Instance 7, and it can be inferred that these are transition activities from “Preparing breakfast” to “Breakfast”.
5. Similarly, investigating Instance 9 shows that Act18 (Toileting) has been predicted with a probability of 0.52 and Act24 (Waking up) with probability of 0.47. Using the domain knowledge, it can be inferred that the user normally goes to the toilet after waking up, so it is a transition between these two activities.
6. Similar transitions can be observed between Act18 and Act 20 (as indicated by Instance 11); however, in Instance 3, it can be clearly seen that Act01 and Act18 have been misclassified with high confidence of 0.98 and therefore, it can be inferred as noise according to the set threshold.

It can be observed that the probability estimates are also helpful in indicating the behavior of the model, in cases where it has overconfidently misclassified an activity (e.g., above a threshold of 0.6) or under-confidently identified a true prediction (e.g., below a threshold of 0.6). If such situations are learned from continuous monitoring, then this behavior can be modeled in decision making. For example, in Table 7, it can be identified that Instance 3 has been misclassified as Act06 overconfidently, but in the case of Instance 6, the medication activity (Act01) has been identified underconfidently.

### 5.3. Identifying Reliable and Non-Reliable Activities for Diabetes Use Case

Based on the results from setting thresholds, interpreting predicted activities and integrating domain knowledge, three categories of instances of an activity have been identified for the diabetes use case: *Reliable_Instances,* which include the actual reliable instances of an activity; *Transitional_Instances,* which include those instances having a confidence score less than α yet are accurate and represent the transition to/from one activity to another; and *Nosiy_Instances,* which include instances that represent noise due to various sources of uncertainty in the dataset.

**Figure 6:**
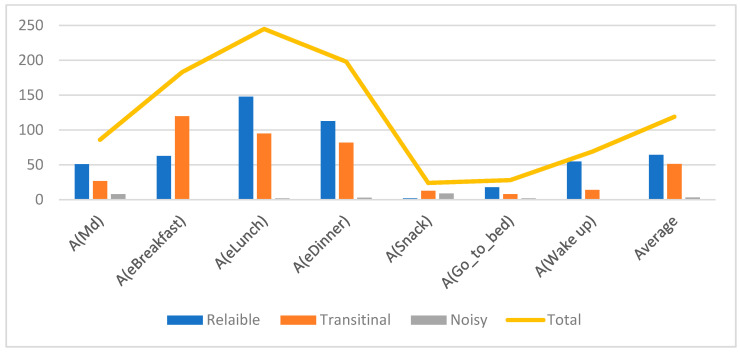
Distribution of reliable, transitional and noisy instances for a diabetes use case.

Figure 6 presents an analysis of the resultant distribution of instances based on the three categories. It can be observed that the majority of the instances are identified as reliable except for the Eat a Snack activity. This is because the majority of the instances of the Snack activity have been misclassified and should not be used in the decision-making process. The yellow line highlights the total number of instances that belong to each activity. The average distribution of all activities indicates that approximately 53.38 percent are reliable, 42.59 percent are transitional and only 4.03 percent are noise.

These results indicate that the application of the CRF model successfully differentiates reliable activities where an action can be taken with minimal risk. Additionally, only 4.03 percent of overall instances are forwarded to the human operator to investigate; therefore, the human operator is not overloaded with information.

### 5.4. Recommended Actions Rules for the Diabetes Use Case

This section introduces the action rules and how these rules can be used to recommend action considering the three types of categories identified as reliable, transitional and noise. For the purposes of this study, the first author of the paper performed the role of an expert to recommend action.

#### 5.4.1. Notations for Action Rules

The confidence level, which has been indicated with α as the threshold and set as 0.8, is subjective, and an alternative value can be chosen depending on the requirement of the real application domain. *β* is the lower range of confidence, and any instance predicted equal or less than that will not be taken into consideration for decision making directly. Likewise, it is also a subjective score and has been set to 0.1 (10 percent) in the proposed scenario.

A(Md) is the medication (Md) activity and represents the treatment activity (A) for the diabetes use case. Similarly, A(Snack) is Act08 and represents a frequent eating case as a symptom of diabetes. A(eBreakfast, elunch, edinner) represents eating € activities as breakfast, lunch and dinner. A(Go_bed) and A(Wake_up) represent Act24 (Go to bed) and wake up activities, respectively. SA(A1,A2,A3…An) are sequential activities (SA), and these are activities which either come before or after the actual activity. For example, medication activity may occur before watching TV and after dinner. In this case, these two activities are declared as SA activities. Max_n and Min_n are the maximum or minimum number of instances that might be associated with a confidence score. For example, six instances of medication activity mean (6∗5 s=30 s) as a 5-s window is used for segmentation. Pb is the predicted probability of an instance of an activity, for example, medication (Pb=0.8) means that predicted activity is medication and Pb is its associated probability. 

#### 5.4.2. List of Actions

Various actions have been developed depending on the predictions, their probabilities and the contextual information and are shown in Table 9. These actions are recommended to reduce the impact of incorrect predictions made by the CRF technique.

#### 5.4.3. Generating Action Rules 

An action rule defines the specific actions to take when certain conditions are met. A basic action rule uses an IF-THEN statement to associate a condition (if) with an action (then). The rules presented in this section integrate the confidence scores of the CRF with domain knowledge that provides some conditions (e.g., the type of activity and its sequence order) in the antecedent part of the rule. The consequent part of the rule comprises a recommended action. The rule states which action to perform when the conditions (could be a combination of several conditions) are true. A list of some action rules is given below for the purpose of illustration. 

**Rule 1:**

IF the predicted instance as a probability distribution {(A(Md), α1), (A(Snack), α2), (A(eBreakfast, elunch, edinner), α3), (A(Go_bed), α4), (A(wake_up), α5), and if αi (i=1,…, 5)} is equal to or greater than α, THEN the corresponding activity is declared as reliable.

THEN Action(Automata) and Action(Disable Reminder)

Description of the rule: the sum of α from all the treatment activities must be equal to 1. If α is less than the threshold (0.8 in this case), the rule will not be executed and the condition will be passed to the next rule.

**Rule 2:**

IF the predicted instance as a probability distribution {(A(Md), α1), (A(Snack), α2), (A(eBreakfast, elunch, edinner), α3), (A(Go_bed), α4), (

A(wake_up), α5), and if αi (i=1,…, 5)} is less than α and greater than *β* and Pb of $SA\left(a_{1},a_{2},\;a_{3}\ldots a_{n}\right)$) is greater than *β*, THEN the corresponding activity is declared as (transition, Pb).

Action: Action(abstain)

**Description of the rule:** the rule identifieS the transition activities that are less than the threshold (0.8) and greater than *β* (Pb less than 0.01 is considered insignificant ), such that they cannot be declared as reliable. In this case, the rule will declare the activity as transitional with its corresponding probability estimate. This rule is generated to separate transitional activities from reliable activities. 

**Rule2A:** IF the predicted instance as a probability distribution:$\;\{(SA\left(A_{1},\alpha_{1}\;\right)$, $SA\left(A_{2},\alpha_{2}\right)$, $SA\left(A_{3},\alpha_{3}\right)$….$SA\left(A_{n},\alpha_{n}\;\right)$ is greater than {(A(Md), α1), (

A(Snack), α2), (A(eBreakfast, elunch, edinner), α3), (A(Go_bed), α4), (A(wake_up), α5), and if αi (i=1,…, 5)} THEN the corresponding activity is declared as true (Pb).

**Action:***Action(abstain)*

**Description of the rule:** The same condition applies as that of Rule 2 except that only transitional activities (SA) are displayed where the probability is greater than any treatment activity and its associated probability.

**Rule 3:** IF the predicted instance as a probability distribution $\{(SA\left(A_{1},\alpha_{1}\;\right)$, $SA\left(A_{2},\alpha_{2}\;\right)$, $SA\left(A_{3},\alpha_{3}\right)$….$SA\left(A_{n},\alpha_{n}\;\right)\;is\;followed\;by\;\left(Min\_5\right)$ and probability distribution {(A(Md), α1), (A(Snack), α2), (A(eBreakfast, elunch, edinner), α3), (A(Go_bed), α4), (A(wake_up), α5), and if αi (i=1,…, 5))} is greater than α, THEN corresponding activity is declared as reliable.

Action: Action(automat) and Action(Disable reminder)

**Description of the rule:** This rule is developed to ensure that individual reliable instances (above α) will only be considered reliable if followed by at least six (Min_5 = 3- seconds) instances. This rule ensures the individual reliable instances (that might occur due to noise in the data) are filtered out. 

**Rule 4.** IF the predicted instance as a probability distribution:$\;\{(SA\left(A_{1},\alpha_{1}\;\right)$, $SA\left(\;A_{2},\alpha_{2}\right)$, $SA\left(\;A_{3},\alpha_{3}\;\right)$…. $SA\left(\;A_{n},\alpha_{n}\;\right)\;is\;not\;followed\;by\;\left(Min\_5\right)$ and probability distribution {(A(Md), α1), (A(Snack), α2), (A(eBreakfast, elunch, edinner), α3), (A(Go_bed), α4), (A(wake_up), α5), and if αi (i=1,…, 5))} is greater than α, THEN the corresponding activity is declared as unreliable.

$$\mathrm{Action}:\;Action\left(Send_{reminder}and\;Confirm\_activity\right)$$

**Description of the rule:** the description is similar to that of rule 3, except that it identifies individual reliable instances if they are not followed by six reliable instances, since they will be declared as noise. 

**Rule 5.** IF the predicted instance as a probability distribution {(A(Md), α1), (A(Snack), α2), (A(eBreakfast, elunch, edinner), α3), (A(Go_bed), α4), (A(wake_up), α5), and if αi (i=1,…, 5))}. is not equal to Rule 1, Rule 2, Rule 3 and Rule 4, THEN the corresponding activity is declared as noise.

Action: Action(Human_intervention)

**Description of the rule:** Any instances that do not fulfill conditions described in rules 1, 2, 3 and 4 will be executed using rule 5, and the assumption is that such instances are noisy.

The above action rules provide a mechanism to execute the actions either automatically or via a human operator as a recommendation.

## 6. IoT-DM Framework Results Analysis

To compare and evaluate the overall results of the IoT-DM framework, a traditional activity-monitoring framework for diabetes has been considered; this is presented in [67]. In this study, for the diabetes use case, it was observed that the model has inaccurately identified the inhabitant as having symptoms of diabetes for 3 days with an accuracy of 83.4%. For example, the proposed model misclassified the “eating” activity as “idle”, and as a result of this misclassification, 17.6 percent of depression instances were incorrectly identified for the user. This traditional framework lacks the ability to identify the reason behind misclassified instances of the diabetic activity. Although the framework enables automation, there is no method to identify only reliable instances of an activity. Furthermore, human operators deal with crisp output and therefore are not able to understand a model’s behavior, which is essential for taking critical actions. The lack of aforementioned capabilities in the traditional framework will mislead the medical expert into taking inappropriate actions as it does not provide any mechanism to identify risk in the decision-making process. On the other hand, the proposed IoT risk-based framework provides several metrics to avoid taking inappropriate actions for a similar diabetes use case, as shown in Table 10. The framework enables decision makers to deploy action in a cost-sensitive manner and interpret the model predictions to understand why the model is correct or incorrect about its predictions. Furthermore, it also provides a method for humans in the loop to rely on reliable instances of activity for taking an action. The necessary details of these matrices have been discussed and demonstrated in subsections of Section 5 for a diabetes use case. 

## 7. Conclusions and Future Work

Our detailed review of related research has shown that IoT systems can be used for reliable decision making despite the existence of several sources of uncertainty. The output of the literature review has been summarized and presented in the form of risk-based framework for decision making in IoT systems. The particular focus of this paper is on managing the underlying sources of uncertainty in IoT systems during the data analytics process and then integrating domain knowledge with ML outputs. We can then use action rules for recommendation and decision support. It was also observed that the well-calibrated CRF method not only supports the quantification of uncertainty in the predictions of an ML model but also provides a natural way to integrate it with domain knowledge. As a result, the integration of domain knowledge, such as the context of an activity, enables us to identify the real activity performed using a thresholding method. Additionally, if an activity has been predicted below the threshold, it can be explained in human-understandable terms. Subsequently, the decision maker can use the confidence scores to compute the cost associated with a decision. It was also found that the outputs of the ML algorithm can be encoded with rich contextual information to change an uncertainty into an opportunity. The proposed IF-THEN action rules were found to be useful for adding semantics to the predictions, which is usually required in intelligent ubiquitous applications, such as HAR. The results from the framework indicate that raw sensor data was converted into meaningful actions despite several sources of uncertainty. It was demonstrated that in the diabetes scenario the framework identified 53.38 percent as reliable instances, 42.59 percent as transitional and 4.03 percent as noisy instances. It was also shown that actions are not taken in isolation but rather are tightly coupled with ML outputs and domain information in the consequent part of the rules. To reduce the impact of inappropriate actions, the framework has included human involvement in cases of high-risk decisions. The comparison of the proposed framework to an existing approach highlighted the key metrics that make decision-making more rational and transparent.

The work in this paper is an initial attempt to survey the current literature and improve decision-making in IoT setups and its scope is limited in a few aspects. Firstly, the approach integrates rich domain knowledge in a simplified way and lacks the use of formal knowledge representation methods, such as the development of an ontology. Secondly, currently, the rules are generated in a static way which might not be suitable when high levels of dynamicity and scalability are required. Additionally, the generated rules are not capable of capturing fuzziness in the decision making. Therefore, in future work, we aim to apply fuzzy rules to address this shortcoming. We also aim to evaluate the framework within different domains and with more heterogeneous datasets to further establish the viability of the framework for IoT systems.

## Figures and Tables

**Table 1:** Mapping of the IoT-DM Framework components to ITU–T reference architecture layers.

IoT-DM Framework Layers	ITU–T Layers
Action and Decision Alternative application and interface for human operator.	Application Layer
Generic Capabilities: Modules such as User Profile, Domain knowledge and Data Processing and submodule Model Selection. Specific Capabilities: Sub modules of Risk-Based Analytics Model Calibration, Confidence Scores, Domain Knowledge, Action Rules and Action Classification.	Service Support and Application support Layer(Generic Capabilities and Gateway Capabilities )
Communication management for Data Acquisition Data Processing and Risk-Based Analytics Module	Network Layer
Data Acquisition (Environmental and wearable sensors) and Actuator(Reminder)	Device Layer (Device Capabilities and Gateway Capabilities )
No Direct mapping	Management Capabilities
No Direct mapping	Security Capabilities

**Table 2:** Activities and number of instances for each activity recorded in the UCAmI Cup dataset.

ID	Name	Instances	ID	Name	Instances
Act01	Take Medication	240	Act13	Leave smart lab	80
Act02	Prepare Breakfast	390	Act14	Visitor to smart lab	14
Act03	Prepare lunch	984	Act15	Put waste in the bin	298
Act04	Prepare dinner	402	Act16	Wash hands	144
Act05	Breakfast	588	Act17	Brush teeth	540
Act06	Lunch	738	Act18	Use the toilet	144
Act07	Dinner	582	Act19	Wash dishes	96
Act08	Eat a snack	78	Act20	Put washing in machine	96
Act09	Watch TV	642	Act21	Work at the table	379
Act10	Enter smart lab	97	Act22	Dressing	432
Act11	Play a videogame	180	Act23	Go to bed	92
Act12	Relax on the sofa	859	Act24	Wake up	222

**Table 3:** List of selected features using the SFS.

Garbage-CAN (GC)	Laundry Basket (LB)	Medicine Box (MB)	Toothbrush (TB)	Bathroom Tap (BT)	SM1	SM3	SM4
TV-Controller (TC)	Fridge	Wardrobe Door (WD)	Water Bottle (WB)	Entrance Door (ED)	Bed	Time	

**Table 4:** Selected activities from the UCAmI dataset for a diabetes use case.

Symptoms and Treatment of Diabetes	UCAmI Activities
Medication	Act01 (take medication)
Frequent eating	Act08 (eat a snack)
Increased hunger	Act05 (breakfast), Act06 (lunch) and Act07 (dinner)
Fatigue (frequent sleeping)	Act23 (waking up) and Act24 (sleeping)
Increased urination (frequent toileting)	Act18 (use the toilet)

**Table 5:** Examples of instances predicted to be medication activity with their confidence scores.

Actual	Predicted	Act01	Act06	Act07	Act17	Act19	Confidence Status
Act01	Act01	0.88	0	0.07	0	0.01	Above Threshold
Act01	Act01	2.51	0	0	0.1	0.38	Above Threshold
Act01	Act01	0.98	0	0	0	0	Above Threshold
Act01	Act01	0.99	0	0	0	0	Above Threshold
Act01	Act01	0.5	0	0.39	0.07	0	Above Threshold
Act01	Act01	0.61	0	0.38	0	0	Above Threshold
Act01	Act01	0.99	0	0	0	0	Above Threshold
Act01	Act06	0.3	0.69	0	0	0	Above Threshold

**Table 6:** Examples of instances predicted to be medication activity with their associated costs.

Actual	Predicted	Act01	Act06	Act07	Act17	Act19	Cost
Act01	Act01	0.88	0	0.07	0	0.01	0.8
Act01	Act01	2.51	0	0	0.1	0.38	−0.29
Act01	Act01	0.98	0	0	0	0	0.12
Act01	Act01	0.99	0	0	0	0	0.13
Act01	Act01	0.5	0	0.39	0.07	0	0.3
Act01	Act01	0.61	0	0.38	0	0	−0.19
Act01	Act01	0.99	0	0	0	0	0.13
Act01	Act06	0.3	0.69	0	0	0	−0.5

**Table 7:** Examples of instances predicted to be medication activity with their interpretations.

Actual	Predicted	Act01	Act06	Act07	Act17	Act19	Interpretations
Act01	Act01	0.88	0	0.07	0	0.01	Probability of medication activity is 0.88, while probability of dinner activity is 0.07
Act01	Act01	0.51	0	0	0	0.10	Probability of medication activity is 0.51, while probability of washing dishes activity is 0.10
Act01	Act01	0.50	0	0.39	0.07	0	Probability of medication activity is 0.50, while probability of dinner activity is 0.39
Act01	Act06	0.30	0.69	0	0	0	Probability of medication activity is 0.30, while probability of lunch activity is 0.69.

**Table 8:** An example list of activities and their status based on the domain knowledge.

No	Actual	Predicted	Act01	Act02	Act05	Act06	Act07	Act08	Act17	Act18	Act19	Act20	Act22	Act24	Status
1	Act01	Act01	0.97	0	0	0	0.02	0	0	0	0	0	0	0	Reliable
2	Act01	Act19	0.39	0	0	0	0	0	0	0	0.6	0	0	0	Transitional
3	Act01	Act06	0.01	0	0	0.98	0	0	0	0	0	0	0	0	Noise
4	Act01	Act01	0.99	0	0	0	0	0	0	0	0	0	0	0	Reliable
5	Act01	Act01	0.88	0	0	0	0.07	0	0	0	0.01	0	0	0	Reliable
6	Act01	Act01	0.52	0	0	0	0	0	0.1	0	0.38	0	0	0	Transitional
7	Act05	Act02	0	0.52	0.47	0	0	0	0	0	0	0	0	0	Transitional
8	Act05	Act05	0	0.08	0.83	0	0	0	0.08	0	0	0	0	0	Reliable
9	Act018	Act18	0	0	0	0	0	0	0	0.52	0			0.47	Transitional
10	Act018	Act17	0	0	0	0	0	0	0.95	0.95	0	0	0.02	0	Noise
11	Act018	Act20	0	0	0	0	0	0.21		0.07	0	0.71	0	0	Transitional

**Table 9:** List of recommended actions for diabetes use case and their descriptions.

Actions	Description
Action(automate)	Automate actions predicted equal or greater than the high threshold (no human intervention).
Action(Disable or Enable Reminder)	If required enable the reminder service for the user.
$Action\left(Send_{reminder}and\;Confirm\_activity\right)$	If an activity is not predicted with required confidence, then ask the user to confirm if an activity has been taken place or not.
Action(abstain)	This action involves “wait and see” and abstain from an action.
Action(Human_intervention)	This involves human intervention and the situation will be assessed using human judgment.
Action(raise_Alarm)	If an emergency situation arises, e.g., the user does not take medication continuously for few days.

**Table 10:** Comparison of the proposed risk-based IoT framework with the traditional framework.

	Accuracy	Ability to Make Decision Making Cost Sensitive	Interpretability	Misclassification Reason	Confidence of Output	Human Investigation	Automation
Proposed Framework	Yes	Yes	Yes	Yes	Yes	Yes	Yes
Traditional Framework	Yes	No	No	No	No	No	Yes

## Data Availability

The UCAmI dataset analysed in this study is available online at https://drive.google.com/drive/folders/1Ntu2DfQbHqsCpdHSnXVK6I6eJe7qpffk (accessed on 30 June 2021).
